# Ideal adaptive control in biological systems: an analysis of $\mathbb {P}$-invariance and dynamical compensation properties

**DOI:** 10.1186/s12859-024-05718-5

**Published:** 2024-03-04

**Authors:** Akram Ashyani, Yu-Heng Wu, Huan-Wei Hsu, Torbjörn E. M. Nordling

**Affiliations:** Department of Mechanical Engineering, National Cheng Kung University, No. 1 University Rd., Tainan, 701 Taiwan

**Keywords:** Dynamical compensation property, $\mathbb {P}$-invariance, Ordinary differential equations, Adaptive proportional-integral feedback

## Abstract

**Background:**

Dynamical compensation (DC) provides robustness to parameter fluctuations. As an example, DC enables control of the functional mass of endocrine or neuronal tissue essential for controlling blood glucose by insulin through a nonlinear feedback loop. Researchers have shown that DC is related to the structural unidentifiability and the $\mathbb {P}$-invariance property. The $\mathbb {P}$-invariance property is a sufficient and necessary condition for the DC property. DC has been seen in systems with at least three dimensions. In this article, we discuss DC and $\mathbb {P}$-invariance from an adaptive control perspective. An adaptive controller automatically adjusts its parameters to optimise performance, maintain stability, and deal with uncertainties in a system.

**Results:**

We initiate our analysis by introducing a simplified two-dimensional dynamical model with DC, fostering experimentation and understanding of the system’s behavior. We explore the system’s behavior with time-varying input and disturbance signals, with a focus on illustrating the system’s $\mathbb {P}$-invariance properties in phase portraits and step-like response graphs.

**Conclusions:**

We show that DC can be seen as a case of ideal adaptive control since the system is invariant to the compensated parameter.

## Background

Dynamical compensation (DC) implies that the output of a system does not depend on a parameter for any input [1]. For instance, in glucose homeostasis controlled by insulin, despite parameter variations, the glucose response remains identical. Thus DC enables control of glucose despite parameter variation in insulin production. This definition of the DC property is a sufficient condition and implies that the parameter is structurally unidentifiable [2, 3]. Note that it describes a dynamical feature that should not be equated simply to the parameter unidentifiability.

Given that the structural unidentifiability merely serves as a sufficient condition for DC property, it is possible for a system to possess the DC property while remaining structurally identifiable meaning the DC property does not equate directly to structural identifiability [2]. Instead, the DC property characterizes a system’s capability to adjust to alterations in parameters following an adaptation phase. In 2017, a necessary and sufficient condition for the DC property was introduced using equivariances and partial differential equations, denoted as the $\mathbb {P}$-invariance property [4]. The $\mathbb {P}$-invariance property of a parameter means that changing the parameter does not alter the system’s behavior. DC captures the ability of a system to adapt to a parameter change such that the change has no impact on the behaviour.

Adaptive control is a sub-field of control systems engineering focusing on the design and development of control systems that automatically adjust parameters to optimise performance, maintain stability, and deal with uncertainties in the system. Considering that DC makes the system invariant to the compensated parameter, this structure can be used to make an ideal adaptive controller. Surprisingly, DC has not been discussed in the adaptive control literature.

Robustness, which refers to a system’s ability to handle fluctuations, is critical in dynamical systems. Several studies on adaptation and homeostasis have demonstrated the robustness of biological systems, such as the robustness of bacterial chemotaxis [5, 6]. The application of DC and $\mathbb {P}$-invariance properties is also beneficial in epidemiological models [7, 8]. Therefore, the DC property may be included in future robustness research. Karin et al. used the glucose homeostasis model to discuss the robustness and DC property of homeostasis [1].

Several mathematical models based on systems of differential equations have been developed to comprehensively analyze biological observations and identify all possible connections. However, it is often more convenient to work with simpler models with fewer dimensions, as they are easier to interpret and analyze. In this paper, we simplify the original model in Karin et al. to two states, offering insight into the mechanism. Then we include another feedback mechanism to derive an generalized model in “Methods” Section . We began by checking the system’s stability in “Results” section because the system must be stable to check the DC and $\mathbb {P}$-invariance properties. We use the phase portrait approach to verify the system’s stability and obtain some results, for preferred stable situations, to compare the results of the DC and $\mathbb {P}$-invariance properties. Finally, in the “Numerical simulation” section , we considered situations in which the system is stable at desired equilibrium points and demonstrated the impact of adaptive control and $\mathbb {P}$-invariance in the system when it is perturbed.

## Methods

As a starting point, we used the hormonal circuit reactions model stated in Karin et al. [1];

1a $$\begin{aligned} \frac{dy}{dt}&= u_{0} + u(t) - sx(t)y(t), \end{aligned}$$

1b $$\begin{aligned} \frac{dx}{dt}&= pz(t)y(t)-x(t), \end{aligned}$$

1c $$\begin{aligned} \frac{dz}{dt}&= z(t)(y(t)-y_{0}), \end{aligned}$$

where *s* and *p* are the feedback gains of *x*(*t*) and *z*(*t*), respectively. The output variable, *y*(*t*), is a regulated variable that is able to form a feedback loop with *x*(*t*) and *z*(*t*). The regulated variable *y*(*t*) controls the functional mass *z*(*t*) of tissue which secretes hormone *x*(*t*) in this circuit.

Given the resemblance between the DC property and the concept of an adaptive controller, it is logical to design an adaptive controller that incorporates the DC property. Since it is easier to visualize and comprehend a two-dimensional system, we begin by simplifying the model 1, which includes Eqs. 1a–1c. Note that we only seek to preserve the DC property and do not apply model reduction techniques aiming to approximate the dynamics of the system. We then generalize the model to a classical feedback system with adaptive proportional-integral feedback and demonstrate that it possesses the P-invariance property. We also explain the differences between DC and $\mathbb {P}$-invariance property.

**Fig. 1:**
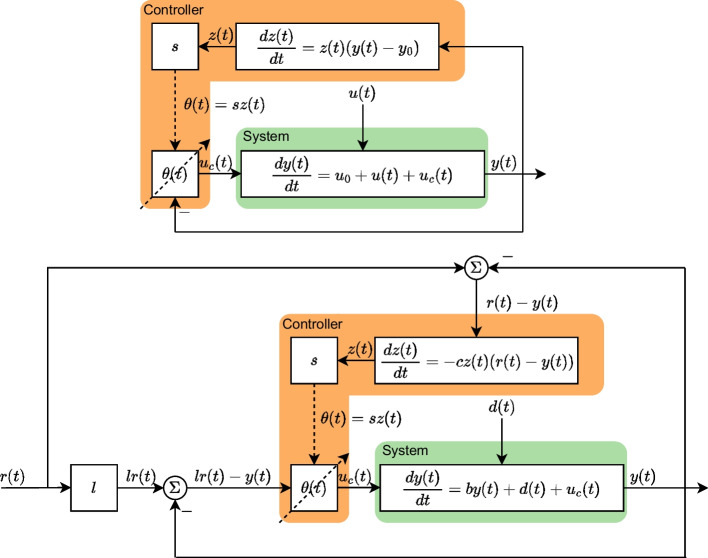
Block diagrams illustrating the simplified model (upper plot) and the adaptive proportional-integral feedback model (lower plot). The upper plot depicts the block diagram of Eq. 2a and 2b, presenting a system (green block) with a negative feedback loop where the controller (orange block) parameter $\theta (t)$ is influenced by *z*(*t*) and *s*. The lower plot represents the block diagram of Eq. 4a and 4b, illustrating the adaptive proportional-integral feedback $sz(t)\big (lr(t)-y(t)\big )$, where *sz*(*t*) is the adaptive proportional-integral gain with two error terms $r(t)-y(t)$ and $lr(t)-y(t)$. The term *d*(*t*) represents the disturbance. Note that *s* has no impact on the control signal due to $\mathbb {P}$-invariance

We simplified the model 1 as,

2a $$\begin{aligned} \frac{dy}{dt}&= u_{0} + u(t) - sz(t)y(t), \end{aligned}$$

2b $$\begin{aligned} \frac{dz}{dt}&= z(t)(y(t)-y_{0}), \end{aligned}$$

where *z*(*t*) is the feedback state, and *y*(*t*) is the output of the system. The block diagram of the simplified model is shown in the upper plot of Fig. 1. We expect that the positive constant *s* has the DC property, meaning that the output *y*(*t*) is invariant to the change of the parameter *s*. Hence we introduce $\tilde{z}(t) = sz(t)$ and substitute *z*(*t*) in Eqs. 2a and 2b with $\tilde{z}(t)$ resulting in

3a $$\begin{aligned} \frac{dy}{dt}&= u_{0} + u(t) - \tilde{z}(t)y(t), \end{aligned}$$

3b $$\begin{aligned} \frac{d\tilde{z}}{dt}&= \tilde{z}(t)(y(t)-y_{0}). \end{aligned}$$

The above Equations show that the output response *y*(*t*) remains the same when the value of *s* changes. This also indicates *s* being unidentifiable and *z*(*t*) being unobservable (verified using STRIKE-GOLDD [9]).

To illustrate DC in a more general two dimensional system, we create an adaptive proportional-integral feedback model

4a $$\begin{aligned} \frac{dy}{dt}&= by(t) + d(t) + sz(t)\big (lr(t)-y(t)\big ) , \end{aligned}$$

4b $$\begin{aligned} \frac{dz}{dt}&= -cz(t)\big (r(t)-y(t)\big ). \end{aligned}$$

This system can be viewed as an open-loop exponential growth system $\frac{dy}{dt} = by(t)$, where *d*(*t*) and *r*(*t*) represent the disturbance and reference input, respectively. The error term is given by $r(t)-y(t)$ and $lr(t)-y(t)$, and the adaptive proportional-integral feedback is $sz(t)\big (lr(t)-y(t)\big )$, where *sz*(*t*) is considered as the adaptive proportional-integral gain. In control theory, a reference input refers to an input signal that guides the system response. Typically, the goal is to make the response *y*(*t*) track the reference input *r*(*t*), such that the error term is zero ($r(t)-y(t)=0$) at the equilibrium point. Furthermore, since we consider this equation in the context of biological phenomena, all parameters are assumed positive. This implies that *b*, *s*, *l*, and *c* are all positive, and for every $t>0$, all *y*(*t*), *z*(*t*), and *r*(*t*) are positive. The block diagram of the adaptive proportional-integral system is illustrated in the lower plot of Fig. 1.

To verify the DC property of our model, the system should be at an equilibrium point before being perturbed by any input. When a system is at an equilibrium point, its value does not change with time. We then triggered the system with a step-like input *r*(*t*) to sketch the response *y*(*t*). We adjust the value of each parameter in Eqs. 4a and 4b to observe how they affect the system. The stability region is discovered by drawing the phase portrait.

As non-native English speakers, we acknowledge the work of OpenAI, L.L.C. in creating GPT3.5 and GPT4, which helped us improve the readability and language of this article.

## Results

Here, we began by checking stability of the system, and then we compared the differences between $\mathbb {P}$-invariance and DC property. Finally, we provided a numerical example to illustrate the result.

### Phase portrait and stability

Our goal is to discover the region of attraction by drawing the phase portrait. By setting the derivative terms in Eqs. 4a and 4b to zero and assuming that the reference $r(t) = r$ and disturbance $d(t) = d$ remain constant, two equilibrium points can be obtained:

5 $$\begin{aligned}&E_1=(y_1,z_1)=\left (-\frac{d}{b},0\right ), \end{aligned}$$

6 $$\begin{aligned}&E_2=(y_2,z_2)=\left (r,\frac{d+br}{sr(1-l)}\right ). \end{aligned}$$

Under the assumption that all parameters are non-negative and the signals *d* and *r* are positive, we note the following: Since $y_1$ is negative in the equilibrium point $E_1$, it is a biologically infeasible state of the system. If $0<l \le 1$, then both $z_2$ and $y_2$ are non-negative, making $E_2$ the equilibrium point of interest. To ensure that $z_2$ remains finite, we first assume $0<l<1$. The local stability of a system can be analyzed by calculating the eigenvalues of the matrix of partial derivatives in equilibrium points, known as the Jacobian matrix. The matrix of partial derivatives for system 4 and its eigenvalues are shown below.

7 $$J(y,z) = \left[ {\begin{array}{*{20}c} {b - sz(t)} & {s(lr - y(t))} \\ {cz(t)} & { - c(r - y(t))} \\ \end{array} } \right],$$

8 $$\lambda (y,z) = \frac{1}{2}(b - sz(t) - c(r - y(t)) \pm \sqrt {(b - sz(t) - c(r - y(t)))^{2} - 4c(z(t)(sr(1 - l)) - b(r - y(t)))} ).$$

The local stability of the system can be analyzed by calculating the eigenvalues at each equilibrium point. When the real parts of the eigenvalues are negative, the equilibrium point is locally stable. The Jacobian matrix is presented in an algebraic structure to calculate the eigenvalues easier when analyzing the local stability of the individual equilibrium point.

*(1) Local stability of* $E_1$: To investigate the local stability around $E_1$, we computed two eigenvalues.

9 $$\lambda _{1} (y_{1} ,z_{1} ) = b,\lambda _{2} (y_{1} ,z_{1} ) = - c(d + br)/b.$$

As $b>0$ and $-c\big (d+br\big )/b<0$ this equilibrium point is a saddle point.

*(2) Local stability of* $E_2$: For the equilibrium point $E_2$ the eigenvalues are

10 $$\begin{aligned} \lambda _{1}=\frac{\tau +\sqrt{\tau ^2-4\delta }}{2}, \hspace{5mm} \lambda _{2}=\frac{\tau -\sqrt{\tau ^2-4\delta }}{2}, \end{aligned}$$

where

11 $$\begin{aligned} \tau&=\textrm{trace} \big (J(y_2,z_2)\big )=\frac{d+blr}{r(l-1)},\end{aligned}$$

12 $$\begin{aligned} \delta&=\det \big (J(y_2,z_2)\big )= c\big (d+br\big ). \end{aligned}$$

Three situations can happen:

1. $$\tau ^2-4\delta =0$$,
2. $$\tau ^2-4\delta <0$$,
3. $$\tau ^2-4\delta >0$$.

In both (1) and (2), stability depends on $\tau$. Hence, if $\tau <0$, then $E_2$ is stable. Based on the assumption that parameters and variables are positive to be meaningful in biology and $l<1$, we have $\tau <0$, which means that $E_2$ is a stable equilibrium point. In situation (3), as $\delta >0$, it will result in $|\tau |>\sqrt{\tau ^2-4\delta }$. Hence, if $\tau <0$, then $E_2$ is stable. Again, based on the assumption of having meaningful parameters in the equilibrium points, $l<1$, the equilibrium $E_2$ is stable. These findings demonstrate that as long as $E_2$ is meaningful in biology, it is a globally stable equilibrium point.

### Influence of the adaptive controller on stability

Our aim is to investigate the influence of the adaptive controller term on the stability of the system. Given a system

13 $$\begin{aligned} \dot{x} = f(x(t),u(t),p),\; y = g(x(t),u(t),p), \; x(0) = \gamma _{p}; \end{aligned}$$

if there exists an equivalent transformation

14a $$\begin{aligned} f(\eta _{p}(x),u,p)&= (\eta _{p})_*(x)f(x,u), \end{aligned}$$

14b $$\begin{aligned} g(\eta _{p}(x),u,p)&= g(x,u), \end{aligned}$$

14c $$\begin{aligned} \eta _{p}(\gamma )&= \gamma _{p}, \end{aligned}$$

where $\eta _*$ denotes the Jacobian matrix of transformation $\eta$, the system has the $\mathbb {P}$-invariance property [4]. By verifying the invariance of the system 4, containing Eqs. 4a, 4b, we were able to discover the parameters that lead to the DC property. We also demonstrated the differences between the definitions of $\mathbb {P}$-invariance and the DC property. Based on the definition of the $\mathbb {P}$-invariance property and the relationship with the DC property, the DC property can be classified as an adaptive control strategy in the system 4.

#### Verification of the $\mathbb {P}$-invariance property

We verify that the system is $\mathbb {P}$-invariant with respect to variation of *s*. In order to verify that the system 4 has the $\mathbb {P}$-invariance property, we introduced $x_{1}(t)$ and $x_{2}(t)$ as two state variables and *y*(*t*) as the output variable of the system. For simplicity, we wrote the system 4 in $x_1$, $x_2$, and *y* as,

15a $$\begin{aligned} \dot{x_{1}}&= -cx_{1}\big (r(t)-x_{2}\big ), \end{aligned}$$

15b $$\begin{aligned} \dot{x_{2}}&= bx_{2} + d(t)+sx_{1}\big (lr(t)-x_{2}\big ), \end{aligned}$$

15c $$\begin{aligned} y&= x_{2}. \end{aligned}$$

The notation here is selected to be identical to the one used by [4]. We considered the possible equivariance $\eta _p(x_1,x_2)=\big (\alpha _p(x_1,x_2), \beta _p(x_1,x_2)\big )$. In this case, the condition $g\big (\eta _p(x),u,p\big )=g(x,u)$ means $\beta _p(x_1,x_2)=x_2$. Therefore, we have $\eta _p(x_1,x_2)=\big (\alpha _p(x_1,x_2), x_2\big )$. Hence,

16 $$\begin{aligned} (\eta _p)_*(x_1,x_2)&\begin{bmatrix} =\frac{\partial \alpha _p}{\partial x_1}(x_1,x_2) &{} \frac{\partial \alpha _p}{\partial x_2}(x_1,x_2)\\ \frac{\partial x_2}{\partial x_1}&{}\frac{\partial x_2}{\partial x_2} \end{bmatrix} =\begin{bmatrix} \frac{\partial \alpha _p}{\partial x_1}(x_1,x_2) &{} \frac{\partial \alpha _p}{\partial x_2}(x_1,x_2)\\ 0&{}1 \end{bmatrix}. \end{aligned}$$

As a result from equation 14a, for parameter *s* our aim is to prove $f(\eta _{s}(x),u,s) = (\eta _{s})_*(x)f(x,u)$. It means:

17 $$\left[ {\begin{array}{*{20}c} { - c\alpha _{s} (x_{1} ,x_{2} )(r(t) - x_{2} )} \\ {bx_{2} + d(t) + s\alpha _{s} (x_{1} ,x_{2} )(lr(t) - x_{2} )} \\ \end{array} } \right] = {\text{ }};\left[ {\begin{array}{*{20}c} {\frac{{\partial \alpha _{s} }}{{\partial x_{1} }}(x_{1} ,x_{2} )} & {\frac{{\partial \alpha _{s} }}{{\partial x_{2} }}(x_{1} ,x_{2} )} \\ 0 & 1 \\ \end{array} } \right]\left[ {\begin{array}{*{20}c} { - cx_{1} (r(t) - x_{2} )} \\ {bx_{2} + d(t) + x_{1} (lr(t) - x_{2} )} \\ \end{array} } \right].$$

Hence:

18 $$\begin{aligned}&-c\alpha _{s}(x_{1},x_{2})\big (r(t)-x_{2}\big ) \nonumber \\&=\frac{\partial \alpha _{s}(x_{1},x_{2})}{\partial x_{1}}\Big (-cx_{1}\big (r(t)-x_{2}\big )\Big ) + \frac{\partial \alpha _{s}(x_{1},x_{2})}{\partial x_{2}}\Big (bx_{2}+d(t)+x_{1}\big (lr(t)-x_{2}\big )\Big ), \end{aligned}$$

19 $$\begin{aligned}&bx_{2}+d(t)+s\alpha _{s}(x_{1},x_{2})\big (lr(t)-x_{2}\big )= bx_{2}+d(t)+x_{1}\big (lr(t)-x_{2}\big ). \end{aligned}$$

By comparing the coefficients in Eq. 18 we have

20 $$\begin{aligned} \frac{\partial \alpha _{s}(x_{1},x_{2})}{\partial x_{1}}&=\frac{\alpha _{s}(x_{1},x_{2})}{x_{1}}, \end{aligned}$$

21 $$\begin{aligned} \frac{\partial \alpha _{s}(x_{1},x_{2})}{\partial x_{2}}&=0. \end{aligned}$$

From Eq. 19 we attained

22 $$\begin{aligned} s\alpha _{s}(x_{1},x_{2})(lr(t)-x_{2})=x_{1}\big (lr(t)-x_{2}\big ), \end{aligned}$$

If $lr(t)-x_{2} \ne 0$, it means

23 $$\begin{aligned} \alpha _{s}(x_{1},x_{2})=\frac{x_1}{s}. \end{aligned}$$

Therefore, there is a Jacobian matrix $\alpha _{s}(x_{1},x_{2})= x_1/s$ that can achieve the transformation of the system. The system could demonstrate $\mathbb {P}$-invariance when *s* is the $\mathbb {P}$-invariance parameter. Next, we investigated whether the parameter *b* has the $\mathbb {P}$-invariance property, meaning that changing *b* will not influence the behavior of *y*(*t*). As a result from equation 14a, for parameter *b* our aim is to prove $f(\eta _{b}(x),u,b) = (\eta _{b})_*(x)f(x,u)$. It means:

24 $$\left[ {\begin{array}{*{20}c} { - c\alpha _{b} (x_{1} ,x_{2} )(r(t) - x_{2} )} \\ {bx_{2} + d(t) + s\alpha _{b} (x_{1} ,x_{2} )(lr(t) - x_{2} )} \\ \end{array} } \right] = {\text{ }}\left[ {\begin{array}{*{20}c} {\frac{{\partial \alpha _{b} }}{{\partial x_{1} }}(x_{1} ,x_{2} )} & {\frac{{\partial \alpha _{b} }}{{\partial x_{2} }}(x_{1} ,x_{2} )} \\ 0 & 1 \\ \end{array} } \right]\left[ {\begin{array}{*{20}c} { - cx_{1} (r(t) - x_{2} )} \\ {x_{2} + d(t) + sx_{1} (lr(t) - x_{2} )} \\ \end{array} } \right].{\text{ }}$$

Thus, it is essential to solve

25 $$\begin{aligned}&- c\alpha _{b}(x_{1},x_{2})\big (r(t)-x_{2}\big ) \nonumber \\&=\frac{\partial {\alpha }_{b}(x_{1},x_{2})}{\partial x_{1}}\Big (-cx_{1}\big (r(t)-x_{2}\big ) \Big ) +\frac{\partial {\alpha }_{b}(x_{1},x_{2})}{\partial x_{2}}\Big (x_{2}+d(t)+sx_{1}\big (lr(t)-x_{2})_{-}\Big ), \end{aligned}$$

26 $$\begin{aligned}&bx_{2}+d(t)+s\alpha _{b}(x_{1},x_{2})\big (lr(t)-x_{2}\big )= x_{2}+d(t)+sx_{1}\big (lr(t)-x_{2}\big ). \end{aligned}$$

By comparing the coefficients in Eq. 25, we have

27 $$\begin{aligned} \frac{\partial \alpha _{b}(x_{1},x_{2})}{\partial x_{1}}&=\frac{\alpha _{b}(x_{1},x_{2})}{x_{1}}, \end{aligned}$$

28 $$\begin{aligned} \frac{\partial \alpha _{b}(x_{1},x_{2})}{\partial x_{2}}&=0. \end{aligned}$$

From Eq. 26 we have

29 $$\begin{aligned} bx_2+s\alpha _{b}(x_{1},x_{2})\big (lr(t)-x_{2}\big )= x_2+sx_{1}\big (lr(t)-x_{2}\big ), \end{aligned}$$

If $lr(t)-x_{2} \ne 0$, it means

30 $$\begin{aligned} \alpha _{b}(x_{1},x_{2})=\frac{x_2+sx_{1}\big (lr(t)-x_{2}\big )-bx_2}{s\big (lr(t)-x_{2}\big )}, \end{aligned}$$

and yields

31 $$\begin{aligned} \frac{\partial \alpha _{b}(x_{1},x_{2})}{\partial x_{1}}&=1, \end{aligned}$$

32 $$\begin{aligned} \frac{\partial \alpha _{b}(x_{1},x_{2})}{\partial x_{2}}&=\frac{(1-b)\big (slr(t)\big )}{\Big (s\big (lr(t)-x_{2}\big )\Big )^2}. \end{aligned}$$

There is no solution of $\alpha _{b}(x_{1},x_{2})$ that can be obtained from Eq. 30 and satisfies the two conditions in Eqs. 27 and 28. This implies that the system is not $\mathbb {P}$-invariant in *b*. Next, we verify that the system is not $\mathbb {P}$-invariant with respect to variation of *c*. In order to verify that the system 4 has the $\mathbb {P}$-invariance property, we introduced $x_{1}(t)$ and $x_{2}(t)$ as two state variables and *z*(*t*) as the output variable of the system. For simplicity, we wrote the system 4 in $x_1$, $x_2$, and *z* as,

33a $$\begin{aligned} \dot{x_{1}}&= bx_{1} + d(t)+sx_{2}\big (lr(t)-x_{1}\big ), \end{aligned}$$

33b $$\begin{aligned} \dot{x_{2}}&= -cx_{2}\big (r(t)-x_{1}\big ), \end{aligned}$$

33c $$\begin{aligned} z&= x_{2}. \end{aligned}$$

The notation here is selected to be identical to the one used by [4].

We considered the possible equivariance $\eta _p(x_1,x_2)=\big (\alpha _p(x_1,x_2), \beta _p(x_1,x_2)\big )$. In this case, the condition $g\big (\eta _p(x),u,p\big )=g(x,u)$ means $\beta _p(x_1,x_2)=x_2$. Therefore, we have $\eta _p(x_1,x_2)=\big (\alpha _p(x_1,x_2), x_2\big )$. Hence,

34 $$\begin{aligned} (\eta _p)_*(x_1,x_2)&= \begin{bmatrix} \frac{\partial \alpha _p}{\partial x_1}(x_1,x_2) &{} \frac{\partial \alpha _p}{\partial x_2}(x_1,x_2)\\ \frac{\partial x_2}{\partial x_1}&{}\frac{\partial x_2}{\partial x_2} \end{bmatrix} =\begin{bmatrix} \frac{\partial \alpha _p}{\partial x_1}(x_1,x_2) &{} \frac{\partial \alpha _p}{\partial x_2}(x_1,x_2)\\ 0&{}1 \end{bmatrix}. \end{aligned}$$

As a result from equation 14a, for parameter *c* our aim is to prove $f(\eta _{c}(x),u,c) = (\eta _{c})_*(x)f(x,u)$. It means:

35 $$\left[ {\begin{array}{*{20}c} {b\alpha _{c} (x_{1} ,x_{2} ) + d(t) + sx_{2} (lr(t) - \alpha _{c} (x_{1} ,x_{2} ))} \\ { - cx_{2} (r(t) - \alpha _{c} (x_{1} ,x_{2} ))} \\ \end{array} } \right]{\text{ }} = \left[ {\begin{array}{*{20}c} {\frac{{\partial \alpha _{c} }}{{\partial x_{1} }}(x_{1} ,x_{2} )} & {\frac{{\partial \alpha _{c} }}{{\partial x_{2} }}(x_{1} ,x_{2} )} \\ 0 & 1 \\ \end{array} } \right]\left[ {\begin{array}{*{20}c} {bx_{1} + d(t) + sx_{2} (lr(t) - x_{1} )} \\ { - x_{2} (r(t) - x_{1} )} \\ \end{array} } \right].$$

Hence:

36 $$\begin{aligned}&b\alpha _{c}(x_{1},x_{2}) + d(t)+sx_{2}\big (lr(t)-\alpha _{c}(x_{1},x_{2})\big ) \nonumber \\&=\frac{\partial \alpha _{c}(x_{1},x_{2})}{\partial x_{1}}\Big (bx_{1} + d(t)+sx_{2}\big (lr(t)-x_{1}\big )\Big ) \end{aligned}$$

37 $$\begin{aligned}&+ \frac{\partial \alpha _{c}(x_{1},x_{2})}{\partial x_{2}}\Big (-x_{2}\big (r(t)-x_{1}\big )\Big ), \end{aligned}$$

38 $$\begin{aligned}&-cx_{2}\big (r(t)-\alpha _{c}(x_{1},x_{2})\big )= -x_{2}\big (r(t)-x_{1}\big ). \end{aligned}$$

By comparing the coefficients in Eq. 37 we have

39 $$\begin{aligned} \frac{\partial \alpha _{c}(x_{1},x_{2})}{\partial x_{1}}&=\frac{b\alpha _{c}(x_{1},x_{2}) + d(t)+sx_{2}\big (lr(t)-\alpha _{c}(x_{1},x_{2})\big )}{bx_{1} + d(t)+sx_{2}\big (lr(t)-x_{1}\big )}, \end{aligned}$$

40 $$\begin{aligned} \frac{\partial \alpha _{c}(x_{1},x_{2})}{\partial x_{2}}&=0. \end{aligned}$$

From Eq. 38 we attained

41 $$\begin{aligned} c x_2 \alpha _{c}(x_{1},x_{2})=x_{2}\big ((c-1)r(t)+x_1), \end{aligned}$$

If $cx_{2} \ne 0$, it means

42 $$\begin{aligned} \alpha _{c}(x_{1},x_{2})=\frac{(c-1)r(t)+x_1}{c}, \end{aligned}$$

and yields

43 $$\begin{aligned} \frac{\partial \alpha _{c}(x_{1},x_{2})}{\partial x_{1}}&=\frac{1}{c}, \end{aligned}$$

44 $$\begin{aligned} \frac{\partial \alpha _{c}(x_{1},x_{2})}{\partial x_{2}}&=0. \end{aligned}$$

There is no solution of $\alpha _{c}(x_{1},x_{2})$ that can be obtained from Eq. 42 and satisfies the two conditions in Eqs. 39 and 40. This implies that the system is not $\mathbb {P}$-invariant in *c*.

#### Verification of the DC property

As a demonstration to show that $\mathbb {P}$-invariance property is more general than DC property, we used the DC property definition by Karin et al. [1] in the system 15, containing Eqs. 15a–15c. By choosing $v_1=s x_1$ and $v_2= x_2$, for $s \ne 0$ we have:

45a $$\begin{aligned} \dot{v}_{1}&= -cv_1\big (r(t)-v_2\big ), \end{aligned}$$

45b $$\begin{aligned} \dot{v}_{2}&= bv_2+d(t)+v_1\big (lr(t)-v_2\big ), \end{aligned}$$

45c $$\begin{aligned} y&=v_2. \end{aligned}$$

Therefore, we can assume $s = 1$, which means DC property with respect to $s \ne 0$.

By choosing $v_1=x_1$ and $v_2= b x_2$, for $s \ne 0$ we have:

46a $$\begin{aligned} \dot{v}_{1}&= -cv_1\big (r(t)-\frac{1}{b}v_2\big ), \end{aligned}$$

46b $$\begin{aligned} \dot{v}_{2}&= bv_2+bd(t)+bv_1\big (lr(t)-\frac{1}{b}v_2\big ), \end{aligned}$$

46c $$\begin{aligned} y&=v_2. \end{aligned}$$

Therefore, we cannot assume $b = 1$ meaning we cannot find a transformation for *b*. Since the DC property is a sufficient condition, we cannot prove that the system has DC property with respect to *b*. However, as $\mathbb {P}$-invariance property is a sufficient and necessary condition for DC, we proved that the system does not have DC for variation in *b*.

By choosing $v_1=c x_1$ and $v_2= x_2$, for $c \ne 0$ we have:

47a $$\begin{aligned} \dot{v}_{1}&= -cv_1\big (r(t)-v_2\big ), \end{aligned}$$

47b $$\begin{aligned} \dot{v}_{2}&= bv_2+d(t)+\frac{s}{c}v_1\big (lr(t)-v_2\big ), \end{aligned}$$

47c $$\begin{aligned} y&=v_2. \end{aligned}$$

Therefore, we cannot assume $c = 1$ meaning we cannot find a transformation for *c*. Since the DC property is a sufficient condition, we cannot prove that the system has DC property with respect to *c*. However, as $\mathbb {P}$-invariance property is a sufficient and necessary condition for DC, we proved that the system does not have DC for variation in *c*.

## Numerical simulation

In this section, we discuss and exemplify the theoretical results of our research by numerical simulations. We verify the phase portrait, influence of the adaptive controller, and the $\mathbb {P}$-invariance property by using step-like responses for input *r*(*t*) and disturbance *d*(*t*). To investigate the $\mathbb {P}$-invariance property with respect to the parameters *s* and *b*, the system 4 is first brought to its equilibrium. Next, by perturbing the system with a step-like response *d*(*t*) and changes in *s* and *b* separately, we check whether it returns to the equilibrium or not.

As analyzed in “Phase portrait and stability” Section , equilibrium point $E_1$ is always a saddle point, and to have stability at equilibrium point $E_2$, the main condition is $0<l<1$. Therefore, we chose initial conditions such that all solutions converge to $E_2$, i.e., a stable equilibrium point.

48 $$\begin{aligned} b=0.3,\hspace{1mm} d(0)=0.01, \hspace{1mm} c=2,\hspace{1mm} r(0)=11,\hspace{1mm} l=0.7, s=0.25 \end{aligned}$$

Hence the system 4 is:

49a $$\begin{aligned} \frac{dy}{dt}&= 0.3y(t) + 0.01 + sz(t)\big (7.7-y(t)\big ) , \end{aligned}$$

49b $$\begin{aligned} \frac{dz}{dt}&= -2z(t)\big (11-y(t)\big ), \end{aligned}$$

We simulated the step-like response with initial input $r(0)=11$ and a single pulse with amplitude 5 from time 0 to 400.

We verified the results with different parameters for *s*, *b* and *c*.

**Fig. 2:**
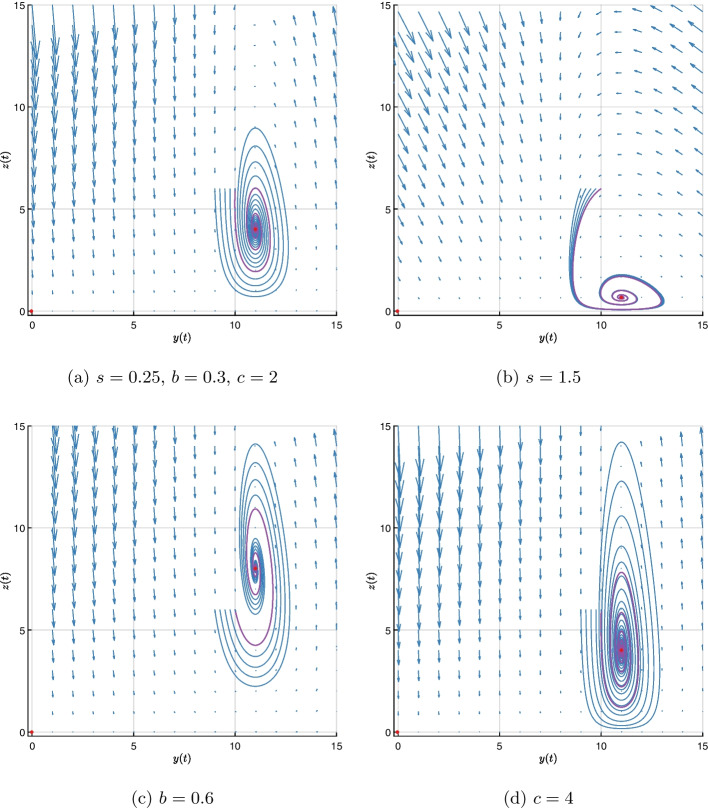
Phase portraits with different values of *s*, *b* and *c* show that the stable equilibrium $E_2$ has almost the same region of attraction in all cases but the trajectories differ. The region of attraction is determined by $E_1$. (a) The phase portrait for parameters 48. (b) The phase portrait for parameters 48, except *s* that is changed from 0.25 to 1.5. (c) The phase portrait for parameters 48, except *b* that is changed from 0.3 to 0.6. (d) The phase portrait for parameters 48, except *c* that is changed from 2 to 4

The phase portrait for the original parameters in 48 with different values of *s*, *b* and *c* is shown in Fig. 2.

For $s=0.25$, two red dots in Fig. 2a represent the equilibrium points $E_1=(-0.033, 0)$ and $E_2=(11.000, 4.012)$, with eigenvalues

50 $$\begin{aligned} \lambda (E_1)=\{0.300, -22.067\},\hspace{5mm} \lambda (E_2)=\{-0.351+ 2.549 i , -0.351- 2.549 i \}. \end{aligned}$$

Since $E_2$ is a stable equilibrium point, all trajectories in its region of attraction approach it.

If we multiply *s* by 6 times ($s=1.5$), we obtain the equilibrium points $E_1=(-0.033, 0)$ and $E_2=(11.000, 0.669)$ in Fig. 2b, with eigenvalues

51 $$\begin{aligned} \lambda (E_1)=\{0.300, -22.067\},\hspace{5mm} \lambda (E_2)=\{-0.351+ 2.549 i , -0.351- 2.549 i \}. \end{aligned}$$

Again, since $E_2$ is a stable equilibrium point, all trajectories in its region of attraction approach it.

In Fig. 2c, we choose $b=0.6$, which is twice as large as the original one, and it alters the equilibrium points to $E_1=(-0.017, 0)$ and $E_2=(11.000, 8.012)$, with eigenvalues

52 $$\begin{aligned} \lambda (E_1)=\{0.6, -22.033\},\hspace{5mm} \lambda (E_2)=\{-0.701+ 3.568 i , -0.701 - 3.568 i \}. \end{aligned}$$

Since $E_2$ is a stable equilibrium point, all trajectories in its region of attraction approach it.

Finally, if we multiply *c* by two ($c=4$), we get the equilibrium points $E_1=(-0.033, 0)$ and $E_2=(11.000, 4.012)$ in Fig. 2d, with eigenvalues

53 $$\begin{aligned} \lambda (E_1)=\{0.300, -44.133\},\hspace{5mm} \lambda (E_2)=\{-0.351+ 3.622 i , -0.351- 3.622 i \}. \end{aligned}$$

All trajectories in the region of attraction approach $E_2$ as it is a stable equilibrium point.

After verifying the stability of the system, we investigated the $\mathbb {P}$-invariance property under different situations.

**Fig. 3:**
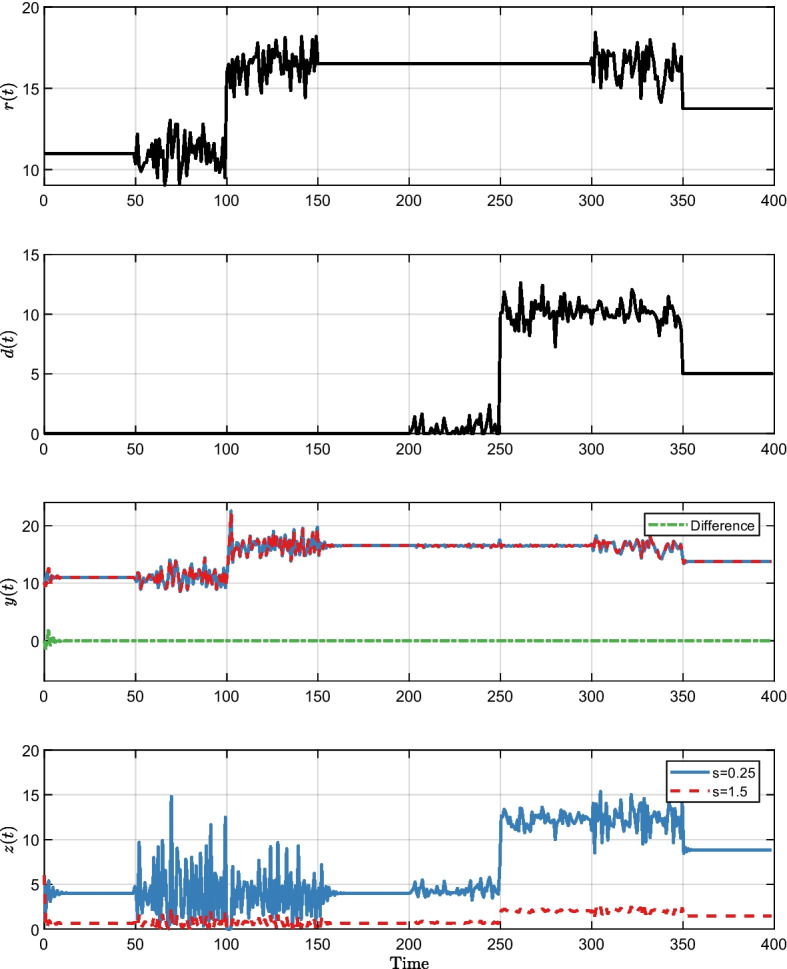
Visualization of the impact of DC and lack thereof on the output using time-varying step-like changes in the reference input *r*(*t*) and disturbance *d*(*t*) in different combinations. Gaussian noise was added to the constant value of *r*(*t*) and *d*(*t*) to demonstrate that the system output remains identical independent of the value of *s* also for complex signals. *y*(*t*) and *z*(*t*) show the comparison of the step response when *s* is 0.25 and 1.5. As we have started *y*(*t*) and *z*(*t*) with a distance from the equilibrium point, it takes time to converge to the stable situation resulting in having some difference between but then the output *y*(*t*) remained identical–the difference (green dashed line) equals zero. A hallmark of the system is $\mathbb {P}$-invariant with regard to *s*

**Fig. 4:**
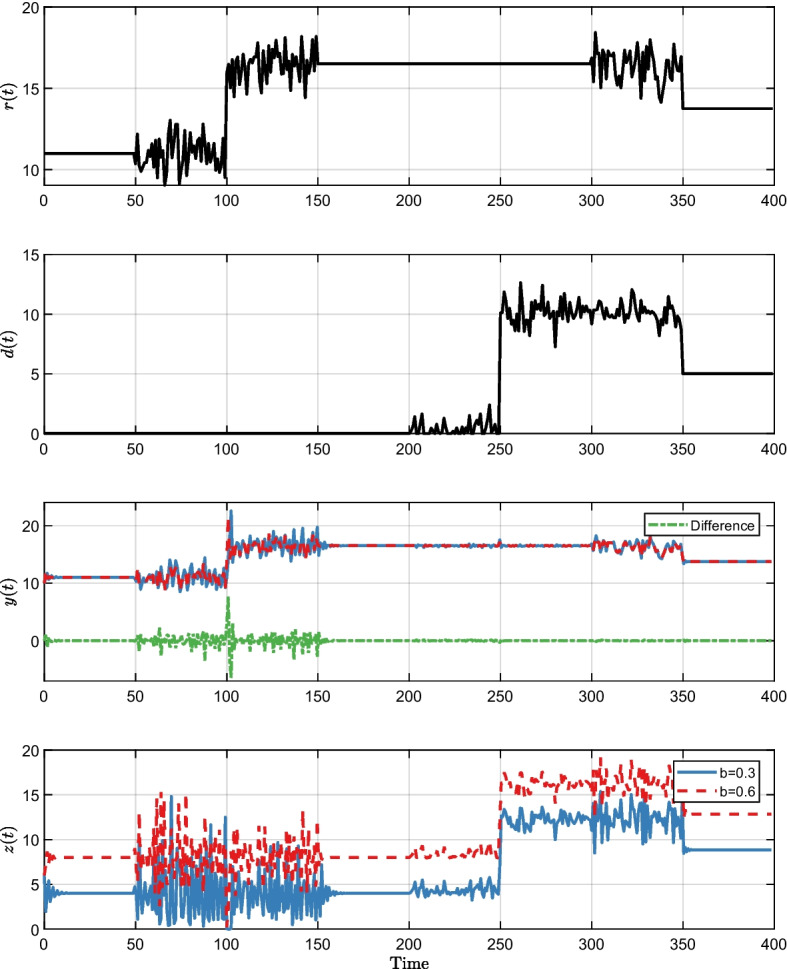
Visualization of the impact of DC and lack thereof on the output using time-varying step-like changes in the reference input *r*(*t*) and disturbance *d*(*t*) in different combinations. Gaussian noise was added to the constant value of *r*(*t*) and *d*(*t*) during certain periods to ensure excitation. *y*(*t*) and *z*(*t*) show the comparison of the step response when *b* is 0.3 and 0.6. The output *y*(*t*) differs and the difference (green dashed line) is non-zero. A hallmark of the system is not being $\mathbb {P}$-invariant with regard to *b*

**Fig. 5:**
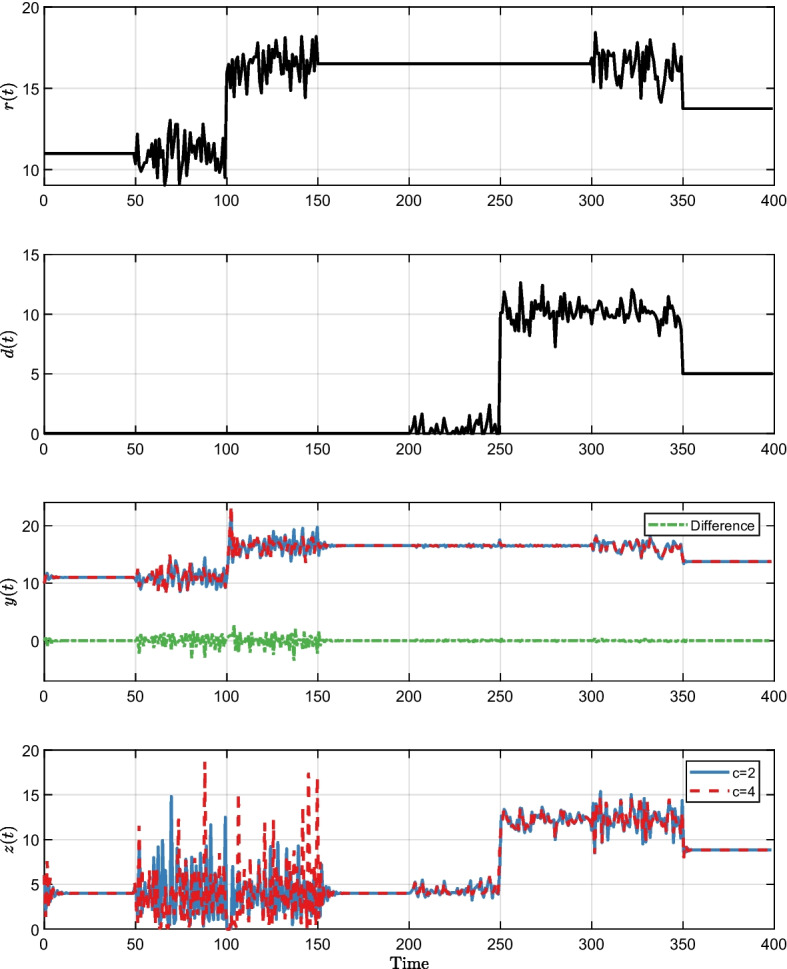
Visualization of the impact of DC and lack thereof on the output using time-varying step-like changes in the reference input *r*(*t*) and disturbance *d*(*t*) in different combinations. Gaussian noise was added to the constant value of *r*(*t*) and *d*(*t*) during certain periods to ensure excitation. *y*(*t*) and *z*(*t*) show the comparison of the step response when *c* is 2 and 4. The output *y*(*t*) differs and the difference (green dashed line) is non-zero. A hallmark of the system is not being $\mathbb {P}$-invariant with regard to *c*

In all Figs. 3, 4 and 5*r*(*t*) and *d*(*t*) are the same and show the time-varying step-like response of the reference input *r*(*t*) and disturbance *d*(*t*). These inputs were also subject to additional noise from a standard normal distribution. We tested different combinations of the reference *r*(*t*) and disturbance *d*(*t*) to exemplify the $\mathbb {P}$-invariance property under different scenarios. Both the input *r*(*t*) and the disturbance *d*(*t*) began with the starting values defined at 48 and remained constant from time 0 until time 50. The input *r*(*t*) changes while disturbance *d*(*t*) remains constant when time is between 50 and 150. Both the input *r*(*t*) and disturbance *d*(*t*) remain constant between time 150 and 200. In the time interval (200, 300), the disturbance *d*(*t*) changes while the input *r*(*t*) remains constant. When time is between (300, 350), both the input *r*(*t*) and disturbance *d*(*t*) change. Finally, both converge to a new amount ($r=13.75, d=5$) and remain constant in the time interval (350, 400).

The purple trajectories in Fig. 2 and the trajectories in Figs. 3, 4, and 5 share identical parameters and initial conditions. The distinguishing factors lie in the inputs *r*(*t*) and *d*(*t*). In Fig. 2, the inputs are set to constant values, mirroring the initial values in Figs. 3, 4, and 5. As a result, we are in a stable situation at the start, however, it may take some time to achieve equilibrium point. In all Figs. 3, 4 and 5*z*(*t*) and *y*(*t*) show the responses to the input and disturbance by *r*(*t*) and *d*(*t*). The green dashed line represents the difference of changes in *y*(*t*), which remains zero when *s* changes, but is non-zero when *b* or *c* changes. This is a consequence of the system having $\mathbb {P}$-invariance for parameter *s*, but not for *b* and *c*.

## Discussion

Our two-state simplified and generalized model based on Karin et al. work [1] preserves the DC property when the parameter *s* is changed. We have demonstrated this using the $\mathbb {P}$-invariance definition by Sontag [4]. With this approach, we have also shown no DC for the parameters *b* and *c*, because the definition of $\mathbb {P}$-invariance is both sufficient and necessary. These results are not only relevant for the theoretical understanding of nonlinear dynamical systems but also open avenues for future research in hybrid systems. Exploring the application of our model in the context of hybrid systems, particularly in synthetic biology, presents a promising direction for further investigation.

Our example system is an exponential growth system with an adaptive proportional-integral controller. Exponential growth is a common feature of many physical systems, such as the early stage of cell growth or disease spread. We have shown that our adaptive proportional integral feedback with DC in the control parameters *s* can stabilize the system and ensure that the response tracks the reference input despite variation in the control parameters. The downside of this is that the closed loop systems behavior cannot be tuned by changing the gain of the controller as customary in e.g. PID-controllers. Moreover, we have demonstrated the stability of the system under a variety of conditions and plotted the phase portrait for a representative example.

## Conclusion

In summary, we have demonstrated an adaptive controller with $\mathbb {P}$-invariance in its parameter *s*. Thus showing that DC can be seen as a case of ideal adaptive control where the system is invariant to the compensated parameter. This can be beneficial for designing robust controllers that can handle environmental fluctuations, in particular in Synthetic biology, as well as for understanding biological systems during modeling and analyzing.

## Data Availability

All data and code for the numerical simulations are available at https://github.com/nordlinglab/DynamicalCompensation-Viz
